# Dedifferentiation and Proliferation of Mammalian Cardiomyocytes

**DOI:** 10.1371/journal.pone.0012559

**Published:** 2010-09-03

**Authors:** Yiqiang Zhang, Tao-Sheng Li, Shuo-Tsan Lee, Kolja A. Wawrowsky, Ke Cheng, Giselle Galang, Konstantinos Malliaras, M. Roselle Abraham, Charles Wang, Eduardo Marbán

**Affiliations:** 1 Heart Institute, Cedars-Sinai Medical Center, Los Angeles, California, United States of America; 2 Department of Medicine, Cedars-Sinai Medical Center, Los Angeles, California, United States of America; 3 Department of Medicine, Division of Cardiology, Johns Hopkins University, Baltimore, Maryland, United States of America; 4 Department of Molecular Medicine, Beckman Research Institute, City of Hope National Medical Center, Duarte, California, United States of America; Instituto de Química - Universidade de São Paulo, Brazil

## Abstract

**Background:**

It has long been thought that mammalian cardiomyocytes are terminally-differentiated and unable to proliferate. However, myocytes in more primitive animals such as zebrafish are able to dedifferentiate and proliferate to regenerate amputated cardiac muscle.

**Methodology/Principal Findings:**

Here we test the hypothesis that mature mammalian cardiomyocytes retain substantial cellular plasticity, including the ability to dedifferentiate, proliferate, and acquire progenitor cell phenotypes. Two complementary methods were used: 1) cardiomyocyte purification from rat hearts, and 2) genetic fate mapping in cardiac explants from bi-transgenic mice. Cardiomyocytes isolated from rodent hearts were purified by multiple centrifugation and Percoll gradient separation steps, and the purity verified by immunostaining and RT-PCR. Within days in culture, purified cardiomyocytes lost their characteristic electrophysiological properties and striations, flattened and began to divide, as confirmed by proliferation markers and BrdU incorporation. Many dedifferentiated cardiomyocytes went on to express the stem cell antigen c-kit, and the early cardiac transcription factors *GATA4* and *Nkx2.5*. Underlying these changes, inhibitory cell cycle molecules were suppressed in myocyte-derived cells (MDCs), while microRNAs known to orchestrate proliferation and pluripotency increased dramatically. Some, but not all, MDCs self-organized into spheres and re-differentiated into myocytes and endothelial cells *in vitro*. Cell fate tracking of cardiomyocytes from 4-OH-Tamoxifen-treated double-transgenic MerCreMer/ZEG mouse hearts revealed that green fluorescent protein (GFP) continues to be expressed in dedifferentiated cardiomyocytes, two-thirds of which were also c-kit^+^.

**Conclusions/Significance:**

Contradicting the prevailing view that they are terminally-differentiated, postnatal mammalian cardiomyocytes are instead capable of substantial plasticity. Dedifferentiation of myocytes facilitates proliferation and confers a degree of stemness, including the expression of c-kit and the capacity for multipotency.

## Introduction

Generations of physicians have been taught that the heart is a static organ, incapable of self-renewal. That dogma has been undermined by the recognition that the adult mammalian heart contains its own reservoir of progenitor (or stem) cells [1]–[5]. Cardiomyocytes in the human heart are renewed throughout life [6]. In contrast to the prevailing view for mammals, hearts from amphibians and zebrafish exhibit surprising potential to regenerate cardiac muscle by partial dedifferentiation [7], [8], and possibly by stem cell-mediated regeneration as well [9]. Dedifferentiation can change the phenotype of specialized cells, rendering them closer to their ancestors with augmented plasticity. For instance, quail pigment cells derived from neural crest can dedifferentiate to become multipotent self-renewing progenitors expressing early neural marker genes Sox10, FoxD3, Pax3 and Slug, and give rise to glial cells and myofibroblasts [10]. Human chondrocytes, epidermal cells, pancreatic beta cells and adipose stromal cells dedifferentiate and exhibit stem cell phenotypes [11]–[15]. Dedifferentiation is a common occurrence in plants; protoplasts from tobacco leaves undergo a transitory phase conferring pluripotentiality, that precedes signal-dependent re-entry into the cell cycle [16].

In adult cardiomyocytes, dedifferentiation has been investigated extensively at the phenotypic level, mainly in non-purified cell culture. In this study, we investigated dedifferentiation of adult atrial and ventricular myocytes and their subsequent phenotype *in vitro* using purified cardiomyocytes as one approach, and a genetic myocyte fate mapping model as a complementary methodology to validate the main conclusions. The salient results are: 1) downregulation of cell cycle inhibitors, 14-3-3, p21 and p53 underlies cardiomyocyte dedifferentiation; 2) dedifferentiated cardiomyocytes divide and generate cardiac progenitor-like cells that express c-kit. The results indicate substantial, unexpected cellular plasticity of postnatal mammalian cardiomyocytes. Formation of new cardiomyocytes may come from both the proliferation of dedifferentiated myocytes without complete reversion to a cardiac progenitor state [17], or by the cardiac differentiation of stem cells (of embryologic or dedifferentiated origin). Several preliminary reports have appeared [18]–[20].

## Results

### Dedifferentiation of Cardiomyocytes

**Figure 1:**
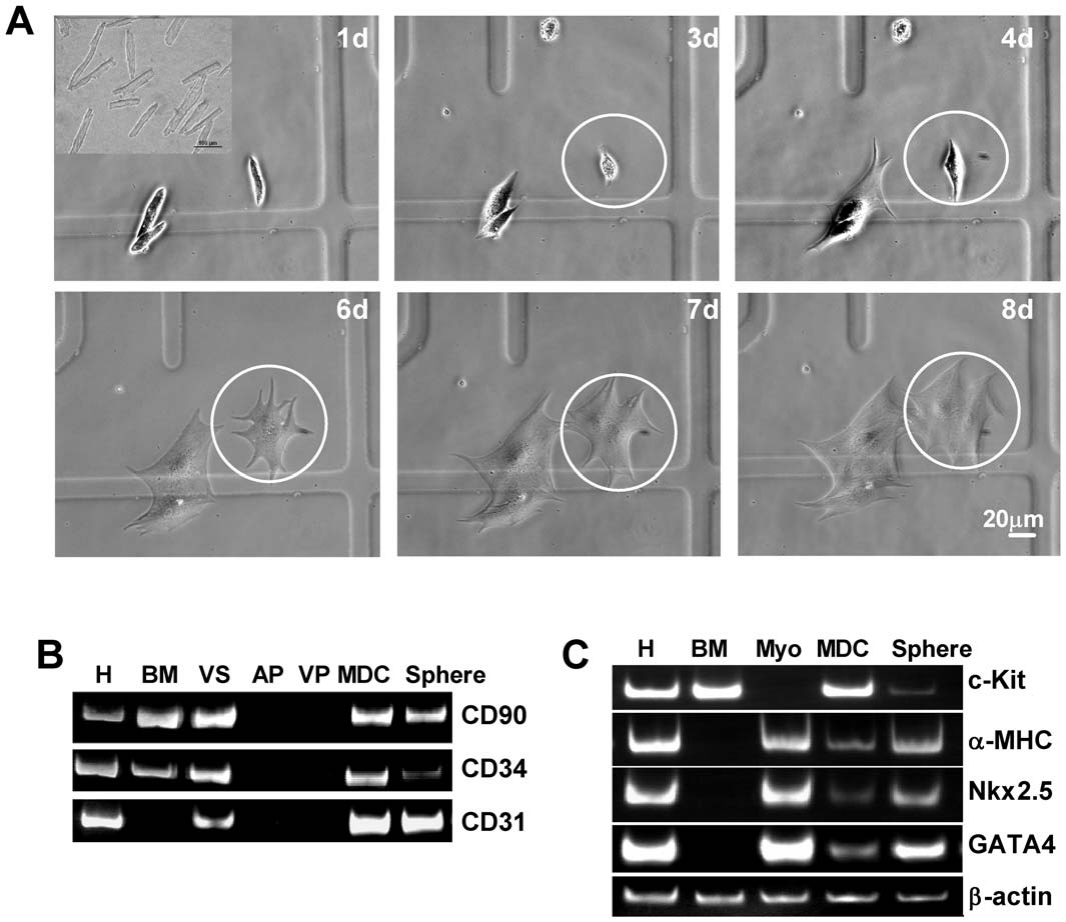
Cardiomyocyte Dedifferentiation. A, Purified myocytes in prolonged culture dedifferentiate and round up and tend to divide. B-C, RT–PCR amplification of cardiac, non-cardiac and stem cell markers. 30–35 3-step cycles were used. H, heart tissue; BM, bone marrow cells; VS, blood vessels; AP and VP, purified atrial and ventricular cardiomyocytes, rep.; Myo, purified myocytes; MDC, myocyte-derived cells; Sphere, MDC-formed spheres.

As cardiac fibroblasts and other non-cardiomyocytes can potentially affect cell cycle activity, it is necessary to eliminate those cells when attempting to specifically study myocytes [21]–[23]. We performed multiple steps in sequential sedimentation, Percoll gradient centrifugation and preplating of rat cardiomyocytes (Figure 1A). Cell preparations were evaluated for purity by the expression of cardiac myofilament proteins as affirmative markers, as well as for c-kit or other non-cardiomyocyte antigens as negative markers [2], [3], [24]. As an alternative to flow cytometry, which is not well-suited for cells as large as adult cardiomyocytes (usual maximal nozzle size is 100 µm) [25], we employed high-density tile scanning confocal microscopy: myocyte preparations of more than 100,000 cells were cyto-spun onto 22 mm culture glasses. No cells expressing c-kit (a resident cardiac stem cell marker), CD31 (PECAM, an endothelial cell marker), CD34 (an endothelial progenitor cell marker) or CD90 (a mesenchymal stem cell marker) were observed in purified cardiomyocyte samples containing as many as 500,000 counted cells (Figure S1) [2]. Transcripts for various non-myocyte antigens were likewise undetectable in purified cardiomyocytes (Figures 1B and 1C). Thus, by tile scanning microscopy, we can set an upper limit for non-myocyte contamination of 1 in 500,000 cells in the starting myocyte cultures. The PCR confirms the purity, albeit with lower sensitivity (Figure S2).

**Figure 2:**
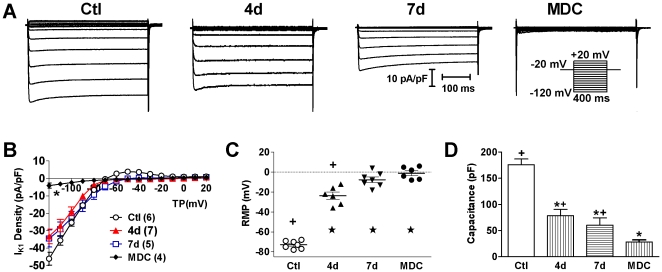
Electrophysiological Dedifferentiation of Cardiomyocytes. A, Example recordings of inward rectifier potassium current (I_K1_) normalized to cell capacitance in freshly-isolated (Ctl) and 4 d or 7 d cultured myocytes, and in myocyte-derived cells (MDC). Inset is the voltage protocol for I_K1_ recording. B, Current-voltage (I-V) relationship of I_K1_ in Ctl or cultured dedifferentiated myocytes or in MDCs. Cell numbers are denoted in brackets. C, Resting membrane potential (RMP); D, Electrical capacitance as a means to measure cell size. ^*^ *P*<0.001 *vs.* Ctl, ^+^ *P*<0.001 *vs.* MDC.

**Figure 3:**
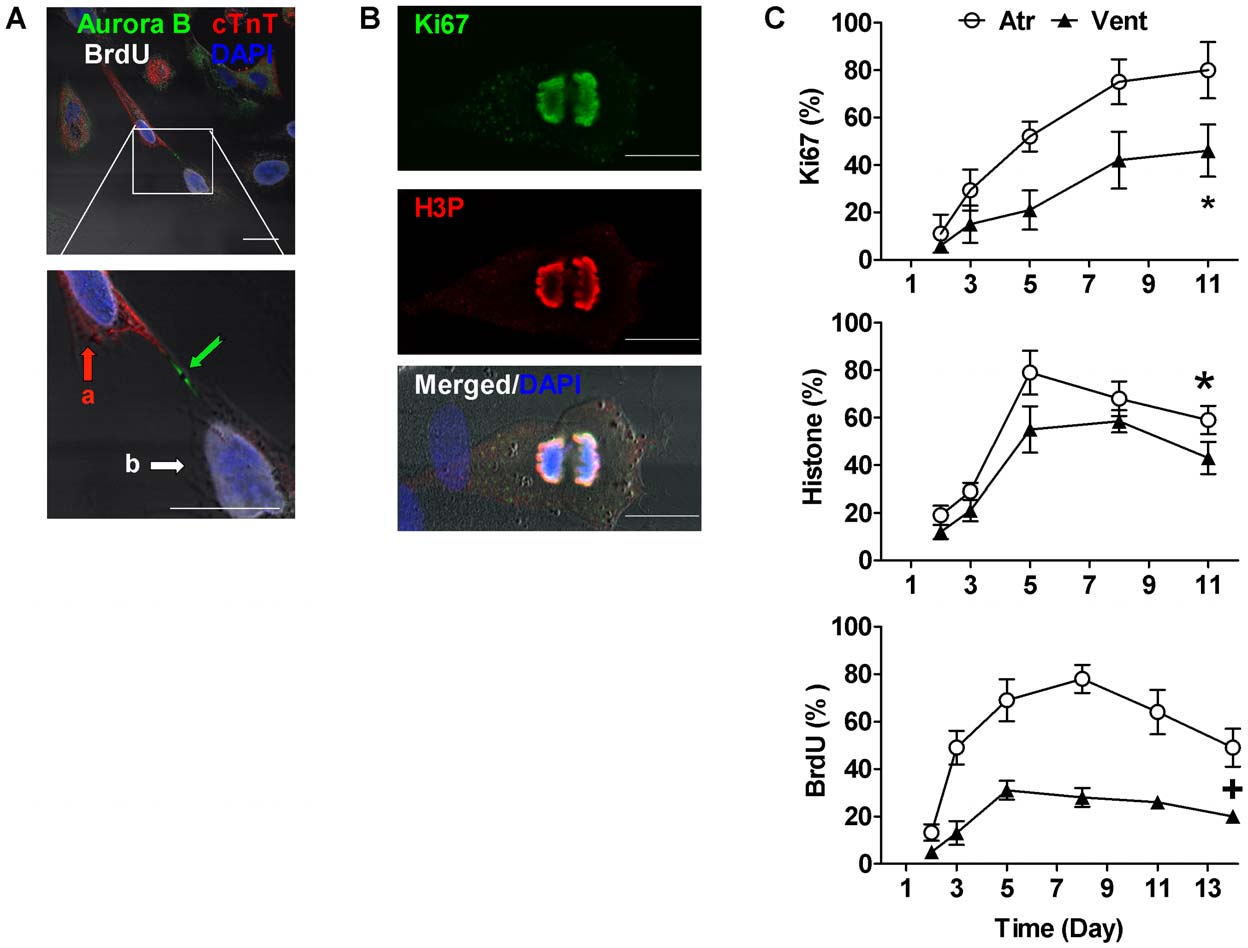
Cell Cycle Progression in Cardiomyocytes. A, Fluorescent immunostaining reveals myocyte dedifferentiated and undergoing cytokinesis as indicated by the expression of Aurora B kinase (Aurora B, green) in the cell cleavage furrow between one cell expressing cTnT (cell a, red) and the other no cTnT (cell b), while both having BrdU incorporated (pseudo-white). Nuclei are stained with DAPI (blue). Scale bars, 20 µm. B, Example immunofluorescent confocal images of tracked myocytes expressing proliferation markers Ki67 (green), phosphor-S10 histone H3 (H3P, red). Scale bars, 20 µm. C, Time course of expression of active cell cycle indices Ki67, H3P, and BrdU incorporation as percentages of myocytes in continuous culture. ^*^ *P*<0.05; ^+^ *P*<0.001 for atrial (Atr) *vs.* ventricular (Vent) myocytes; n = 151–380 cells for different time points.

Individual cardiomyocytes, cultured on grid-marked coverslips continuously in mitogen-rich medium and observed over time, spontaneously flattened and lost their striations (Figure 1A). By 6–8 days, myocytes began to divide while completely losing their distinctive cardiac electrical phenotype: the inward rectifier potassium current (I_K1_) virtually disappeared, resting membrane potential became depolarized, and cells shrank as revealed by decreased electrical capacitance (Figure 2). Some dedifferentiated myocytes no longer expressed myofilament cardiac troponin T (cTnT) (Figure 3A).

### Cell Cycle Progression of Cardiomyocytes

Although dedifferentiation and cell cycle reprogramming have been studied extensively in myocytes from amphibians and zebrafish [26]–[28], the processes are poorly understood in mammalian cardiomyocytes [22], [29]. We analyzed cell cycle progression in this cell culture model by studying the active cell cycle markers Ki67, histone H3 and BrdU incorporation by immunocytochemistry. Ki-67 is a vital molecule for cell proliferation that is expressed in proliferating cells during the active cell cycle, but is absent in resting (G_0_ phase) cells. After 2d in culture, 11±8% and 6±2% of atrial and ventricular myocytes, respectively, re-entered the active cell cycle and expressed Ki-67, with gradually increasing levels, reaching 80±11.9% and 46±11% at 11d for atrial and ventricular myocytes, respectively (*p*<0.001) (Figure 3C). We assessed the proportion of myocytes entering S phase by incubating the cells with BrdU for various periods. Cells in M phase were detected using an antibody against phospho-histone H3 at S10 (H3P). We found a progressive increase in the numbers of BrdU– and H3P– positive cells, reaching a maximum at about 1 week. The proportion of BrdU– and H3P– positive cells was always higher in cultures of atrial myocytes than in those of ventricular myocytes (Figure 3C). Figure 3A shows a dedifferentiated heart cell, still expressing weakly the myofilament protein troponin T (cTnT), budding off a smaller round cell that does not express cTnT, while both have BrdU incorporated and express the mitotic marker Aurora B Kinase in the cell cleavage furrow. Moreover, we also found cells in prophase, anaphase, and telophase (Figure S3), implicating the progression of myocyte proliferation after dedifferentiation.

**Figure 4:**
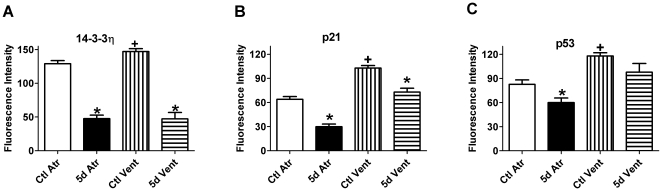
Expression of Regulatory Cell Cycle Factors in Cardiomyocytes. Expression level of inhibitory cell cycle factors 14-3-3η (A), p21 (B) and p53 (C) in freshly isolated (Ctl) or in 5 d cultured atrial (Atr) and ventricular (Vent) myocytes, as measured by the fluorescence intensity in each cell. ^*^ *P*<0.01 *vs.* Ctl of Atr or Vent; ^+^ *P*<0.01 *vs.* Ctl Atr.

To further decipher the mechanisms underlying cell cycle progression and the apparent differences between atrial and ventricular myocytes, we investigated the expression of various critical checkpoint regulators (14-3-3 (YWHAH), p21 and p53) by immunocytochemical detection of cells cultured for 5 days [26]. Expression of the negative cell cycle regulator 14-3-3 has been shown to prevent cell cycle progression and serum-induced proliferation [30], [31]. As predicted, the expression of 14-3-3η, an abundant isoform in the heart, was lower in freshly-isolated atrial myocytes than in ventricular myocytes. Furthermore, on day 5 (the most robust period of cell cycle progression for both types of cells), expression of 14-3-3η was dramatically reduced (Figure 4A). p21 (WAF1/CIP1), a downstream target of 14-3-3 and a key inhibitory factor involving in all phases of the cell cycle [32], was also reduced in cultured dedifferentiating/proliferating myocytes. Its endogenous level was 61% higher in freshly-isolated ventricular myocytes than in atrial myocytes. Furthermore, p53was expressed at much lower levels in fresh atrial myocytes than in ventricular myocytes, and decreased over time in atrial myocytes (but not much in ventricular myocytes) (Figures 4B and 4C). Taken together, the data suggest that the weaker inhibitory signals in atrial myocytes facilitate their progression into the cell cycle; diminution of the inhibitory factors over time in both atrial and ventricular myocytes underlies cell cycle progression and proliferation during dedifferentiation.

### Dedifferentiated Cardiomyocytes Express Cardiac Progenitor Cell Markers

**Figure 5:**
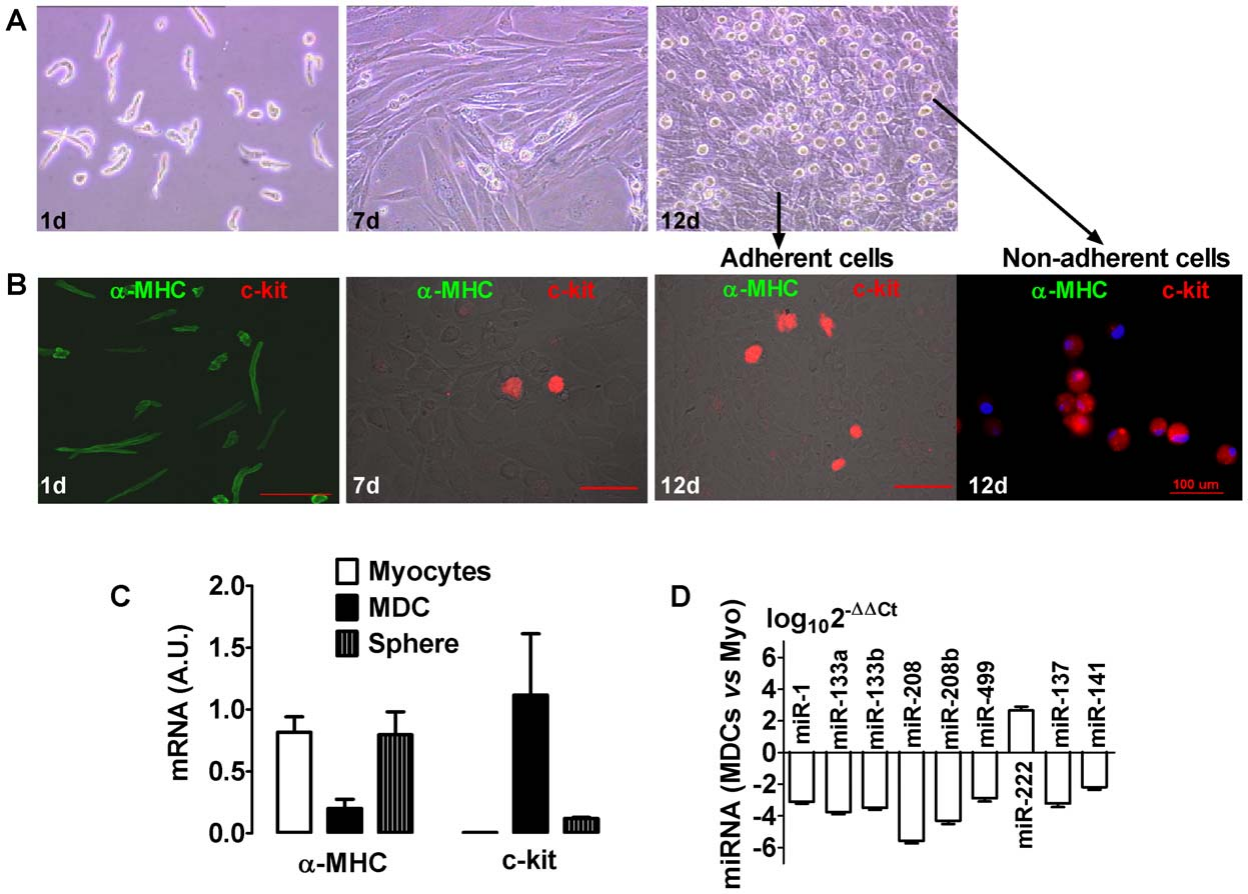
Dedifferentiated Cardiomyocytes Express c-kit. A, Continuous culture of purified myocytes become confluent and give rise to small, phase-bright cells (myocyte-derived cells, MDCs) above the cell layers at about two weeks. Shown are typical images of myocyte cultures at 1 d, 7 d and 12 d. B, Fluorescent immunostaining on cells corresponding to the time shown in (A), revealing the purity of myocyte cultures (1 d) that are positive to α-MHC (green) but negative to c-kit; c-kit (red) emerges in the confluent layer (7 d, 12 d) and in the semi-adherent MDCs (12 d; 61±20%, n = 3 cultures). Scale bars, 100 µm. C, Semi-quantified expression levels of *α-MHC* and *c-kit* detected by RT-PCR in isolate myocytes, non-adherent MDCs (MDC), and MDC-formed spheres (Sphere). Densitometric values were normalized to *β-actin* (arbitrary unit); n = 3. D, Expression levels of regulatory miRNAs in MDCs as compared to freshly isolated myocytes (Myo). Comparative 2^−ΔΔCt^ method was used; n = 3.

Myocytes cultured at the “normal” density of 6,000–9,000 cells/cm^2^, and maintained in continuous (prolonged) culture, went on to yield small, round cells (Figure 5A). Because such cells emerge from confluent cultures, proliferate and have the morphological appearance and size of cardiac progenitor cells [2], [33], we dubbed them myocyte-derived cells (MDCs) and subjected them to further characterization. As dedifferentiation can subsequently contribute to tissue regeneration [10], we investigated the possibility that MDCs may recapitulate at least some of the features of cardiac progenitor cells (CPCs).

As shown in Figure 5, fresh cardiomyocytes are isolated, long striated cells (Figure 5A.*1d*); over time (Figure S4), they become confluent flat cells decorated by a cap of small, round phase-bright myocyte-derived cells (MDCs; Figure 5A.*7d, 12d*). MDCs had undetectable expression of cardiac filament α-MHC, while they stained strongly positive for the stem cell marker c-kit (Figure 5B), although no c-kit was detectable in the founder cardiomyocyte population (Figure 5B.*1d*, Figures 1B–1C, 5C, and Figure S1). In pure myocyte cultures, 2.3–3.1% of dedifferentiated surviving myocytes in monolayers were c-kit^+^ by immunostaining. The semi-adherent cells derived from myocyte culture were also c-kit^+^, albeit weakly (Figure 5B.*12d* “non-adherent cells”). RT-PCR for various transcripts revealed that *α-MHC*, a signature of mature cardiomyocytes, went down in MDCs but rebounded in spheres; conversely, *c-kit* was undetectable in fresh purified cardiomyocytes, rich in MDCs, but sparse in spheres (Figures 1B–1C, and 5C). Sca-1 was non-detectable in MDCs (data not shown). MDCs also expressed *CD90* transcript (for mesenchymal stem cell) as revealed by RT-PCR (Figure 1B) but not at protein level (data not shown).

Additionally, we found drastic changes of regulatory microRNAs (miRNAs) in MDCs as compared to fresh cardiomyocytes. Notably, cardiac-specific miR-1, miR-133, miR-208 and miR-499 were all suppressed by two or more orders of magnitude [34], [35], as were the stemness and cell cycle repressors miR-141 and miR-137 [36]; in contrast, the proliferative miRNAs, miR-222 [37], increased dramatically in MDCs, and miR-221 was undetectable in myocytes but highly expressed in MDCs (Figure 5D). The pattern is consistent with cardiac dedifferentiation, cell cycle progression and re-acquisition of a progenitor cell phenotype; later, MDCs spontaneously re-differentiate as they form spheres, a conjecture consistent with the observed changes in myocardial transcripts *Nkx2.5* and *GATA4* (Figure 1C).

### Re-Differentiation of Myocyte-Derived Cells

**Figure 6:**
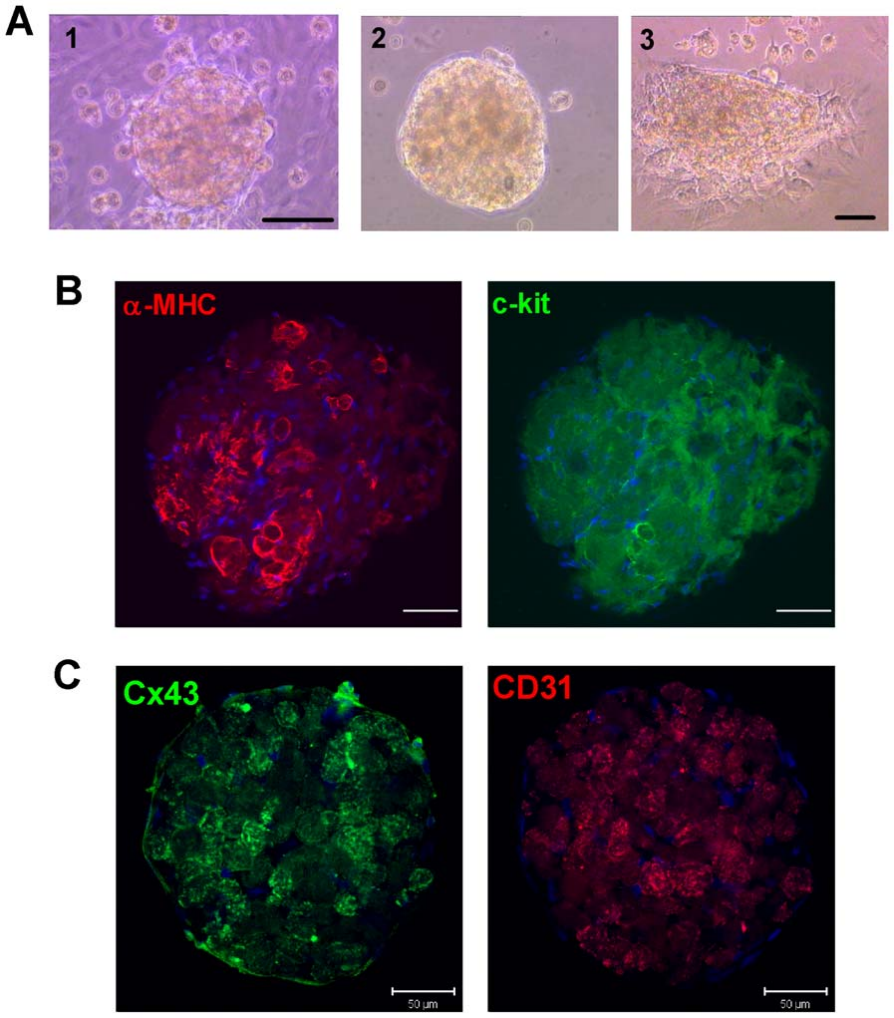
Re-differentiation of Myocyte-Derived Cells (MDCs). A, MDC-formed spheres loosely adhere on the myocyte culture layer (*1*) or detach and become suspended, and eventually beat spontaneously (Supplemental Movies S1–S4); *(2)* Freshly harvested MDC sphere seeded onto culture slide; *(3)* MDC sphere flattened on the culture vessel with cells crawling off the sphere 3 hr after plating. Scale bar, 100 µm. B, Confocal images of fluorescent immunohistochemistry showing the expression of cardiac markers α-MHC (red), and stem cell marker c-kit (green). C, Expression of Cx43 (green) and CD31 (red) in another MDC-formed sphere. Scale bars, 50 µm.

To study the re-differentiation process in more detail, we characterized the properties of MDC-formed spheres. When MDCs above the culture layer become more confluent, 20–40% self-organized into spheres after 3–5 days in continued culture (Fig. 6A). Spheres detached spontaneously (Figure 6A.*2*) and often contracted rhythmically (Figure S4C; Movie S1–S4), signifying competent excitation-contraction coupling. Moreover, confocal imaging revealed that spheres not only express α-MHC (Figure 6B) but also the cardiac gap-junction protein Cx43, as well as the endothelial marker CD31 (Figure 4C). Transduction of MDC-formed spheres with lentivirally-encoded *eGFP* driven by the cardiac *α-MHC* promoter revealed remarkable green fluorescence, in line with their contractile activity and apparent ability to re-differentiate (Figure S5, Movie S5, and Figure 6). One notable feature is the ability of source cardiomyocytes, uncontaminated by endothelial cells or CSCs (Figures 1 and S1), to yield endothelial cells as revealed by the expression of *CD90*, *CD34* and *CD31* (Figures 1B and 6C). This finding manifests the *in vitro* multilineage potential of MDCs.

### Genetic Cell Fate Mapping of Cardiomyocytes

**Figure 7:**
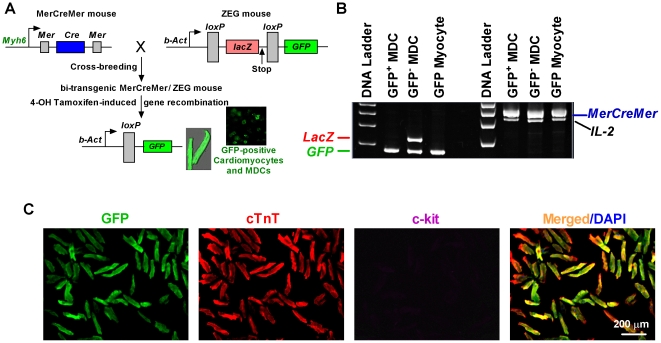
Generation of MerCreMer-Z/EG Bi-Transgenic Mice. A, Cardiomyocytes and their progeny will constitutively express eGFP reporter gene driven by β-actin promoter (*b-Act*) after the excision of floxed *lacZ* mediated by tamoxifen-sensitive *Cre* that is under the control of full length cardiac α-MHC (*MYH6*) promoter. B, PCR detection on transgenes *MerCreMer*, *LacZ*, *GFP*, and internal control (*IL-2*) in isolated GFP myocytes, and GFP^+^- or GFP^−^–MDC demonstrating the cardiac specific-gene recombination induced by tamoxifen. C, Fluorescent immunocytochemistry revealed that cardiomyocytes from 4-OH-Tamoxifen-treated bi-transgenic MerCreMer-Z/EG mice are positive to both GFP (green) and cardiac marker cTnT (red), but not to stem cell marker c-kit. Nuclei were counterstained with DAPI (Blue). Scale bar, 100µm.

The data presented thus far, although highly suggestive, hinge on the purity of the starting preparation of cardiomyocytes. We thus sought independent verification, by genetic fate mapping using double transgenic MerCreMer/ZEG mice, of the notion that adult mammalian cardiomyocytes can dedifferentiate into c-kit^+^ cells [38]. MerCreMer mice carry a fusion transgene of *Cre* recombinase flanked by *Mer* (mutated estrogen receptor ligand-binding domains) that is driven by a cardiac α-MHC promoter (encoded by *Myh6*); thus the Cre recombinase activity is tamoxifen-sensitive and cardiomyocyte-specific [39]. Additionally, ZEG reporter mice carry *lacZ* transgene flanked by *LoxP* sites, followed by stop codons and then *eGFP* gene [40]; therefore, upon excision of the *LoxP* sites and stop codons mediated by Cre recombinase activity, the reporter will switch to GFP (driven by *β-actin* promoter), permanently marking cardiomyocytes and their progeny as GFP-positive (Figure 7A). Cardiomyocytes were identified unambiguously in explants of MerCreMer/ZEG bitransgenic tamoxifen-pulsed mouse hearts by virtue of their co-expression of GFP and cardiac myofilament cTnT (but not c-kit) after *α-MHC*-promoter-driven gene recombination (Fig 7C) [20], [38]. The fidelity of this cardiac *Cre/LoxP* system was also confirmed by genotyping showing that the floxed *LacZ* gene (encoding β-galactosidase) was excised in GFP^+^ myocytes after gene recombination induced by 4-OH tamoxifen (Figure 7B) [20].

**Figure 8:**
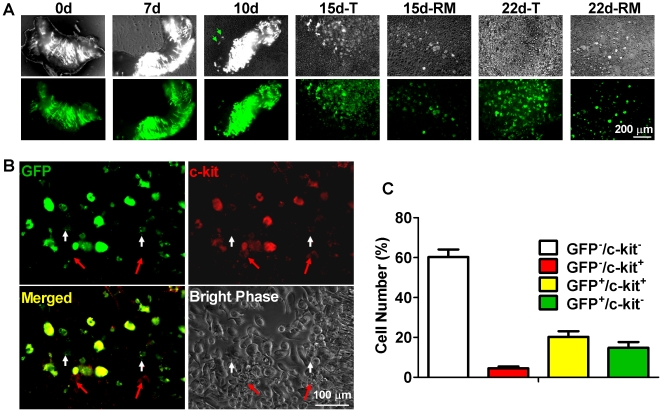
Genetic Cell Fate Mapping of Cardiomyocytes. A, Sample micrographic bright phase (upper panels) and fluorescent images (lower panels) of cardiac tissue cultures at various time points. Green arrows shown at 10 d panel indicate that GFP-positive cardiomyocyte progeny start to migrate off the tissue. T, tissue; RM, remote area. B, Outgrowth cells in remote area showing the expression of GFP (green) and c-kit (red). GFP^+^/c-kit^+^ cells are in yellow color; red arrows indicate the c-kit^+^/GFP^−^ cells which may come from the resident c-kit cells, and white arrows indicate the c-kit^−^/GFP^+^ cells which are GFP myocyte progeny not acquiring the c-kit phenotype yet. Scale bar, 100 µm. C, Microscopy analysis of outgrown cells in random scanning fields 2 microscopic views apart from the MerCreMer/ZEG heart tissue cultures at 3-week using ImageJ software. Mean data was from cultures of 5 MerCreMer-Z/EG hearts; 8–10 cultures and 10 fields in each culture were observed.

To check the myocyte dedifferentiation *ex vivo*, we modified the tissue culture technique to attain better viability of mouse myocytes [4], [20]. As reported [4], [24], small chunks of plated ventricle spontaneously shed “outgrowth” cells. Early on (5 days post-plating), the outgrowth of tamoxifen-pulsed bitransgenic mice contains ∼6% c-kit^+^ cells, but no GFP^+^ cells [20]. After 10 days in culture, however, surviving GFP^+^ cells within the explant and the surrounding outgrowth had begun to round up, giving rise to progeny in which some were weakly c-kit^+^; the changes were unequivocal by 3 weeks in culture (Figure 8), at which time many GFP^+^ cells had become c-kit^+^. We quantified these observations by counting cells in 10 different fields from 8–10 separate outgrowth samples in 2 microscopic views distant from the cardiac explant (Figures 8B–8C). c-kit^+^ cells were either GFP^+^ or GFP^−^, and some GFP^+^ cells lacked c-kit expression; thus, both resident CPCs (GFP^−^) and MDCs (GFP^+^) contribute to the c-kit^+^ cell population [1], [41], [42].

## Discussion

Although the concept of dedifferentiation of a specialized cell precedes the antibiotic era [43], cell fate was generally believed to be unidirectional and irreversible. Mounting evidence now supports the notion that various types of functionally-specialized cells can change their fate under the influence of environmental factors. Examples include amphibian hearts and limbs, and zebrafish hearts, that, upon amputation, regenerate the lost parts by a combination of dedifferentiation [7], [8], [28], [44], activation of stem cells [9], and cell-cycle re-entry without full dedifferentiation [17]. Dedifferentiated myocytes flatten and spread out in culture [45], [46]; sarcomeres become ill-defined and disorganized, and the expression of contractile proteins is dramatically altered [47], [48]. Phenomena akin to *in vitro* dedifferentiation have also been described *in vivo*, in fibrillating atria [49], in chronically-ischemic myocardium, and in the border zone of myocardial infarcts [50]. Such dedifferentiated myocytes are not apoptotic and presumably reflect adaptations to abnormal myocardial stress and/or perfusion [51]. Indeed, dedifferentiated myocytes in continuous culture in the present study were not apoptotic as verified by TUNEL assay (data not shown). In multicellular organisms, dedifferentiation is an important process underlying regeneration and the formation of new stem cell lineages [52]. We have demonstrated that muscle cells from the adult mammalian heart can dedifferentiate and produce cells with antigenic and morphologic features of the cardiac progenitor phenotype.

### The cell culture system

To specifically address the issue of myocyte dedifferentiation and proliferation, we employed multiple centrifugation steps and Percoll gradient separation, resulting in highly-pure myocytes (Figure 1, and Figures S1–S2). This minimizes possible contamination by resident CPCs, and removes nonmyocyte cells that may be capable of transdifferentiation, such as fibroblasts and endothelial cells, as well as any resident CPCs that may have been present in the initial isolates.

Previous work on cardiomyocyte dedifferentiation reported the morphological changes that we describe in early culture [48], but stopped short of observing cell cycle re-entry and acquisition of “stemness”. Those studies used cytosine arabinoside (AraC) or other inhibitors to suppress the growth of nonmyocytes [29], [49], [53], which may also have stopped or delayed cell cycle progression of cardiomyocytes. In pilot studies, we noticed that AraC dramatically suppressed cell cycle progression in atrial myocytes even 3 days or more after its removal from culture medium, and the H3P index was reduced from 75±12% at day 5 to 34±11% (p<0.001) at day 9. In our experiments, the higher Ki67-, BrdU- and H3P-indices as compared to previous studies, might be due to such key differences in culture conditions. In addition, after the removal of dead and detached cells in the first few days of culture, the culture medium was changed much less frequently here than in previous studies. Self-conditioned medium may provide positive feedback for cell cycle progression and transformation of cell fate; we are now exploring this concept. Furthermore, the change of culture medium in previous studies might also remove proliferating myocytes and emerging MDCs that are semi-adherent and exhibit progenitor cell phenotypes.

We noted that, within the first 2–3 days, 20–30% myocytes were lost during the change of medium to remove dead and nonattached cells. The cell culture became stable thereafter; this phenomenon also happened in explant culture of bi-transgenic cardiac tissue (Figure 8A.*0d* and *7d*) and presumably reflects apoptosis of some myocytes in the early period. Therefore, we speculate that dedifferentiation may function as a reprogrammed survival mechanism for stressed cardiomyocytes [54]. Such a mechanism may help rationalize the unusually high prevalence of c-kit^+^ cells in failing human myocardium [55], a setting rich in “hibernating” dedifferentiated myocytes [56].

### Dedifferentiated cardiomyocytes give rise to progenitor cells

Using low-density culture on grid-marked coverslips, and normal dense culture, we were able to characterize dedifferentiation in both single myocytes and in myocyte pools. Most of the cardiomyocytes were in a quiescent G_0_ phase, evidenced by a low Ki67 index even after one day in culture. 14-3-3 and p21 are important inhibitory factors of the cell cycle, and the ablation of the p53–p21 pathway facilitates the generation of induced pluripotent stem (iPS) cells [26], [57]. While we have not excluded the possibility that reactivation of positive regulators of cyclins and cyclin-dependent kinases might contribute to cell cycle re-entry in adult myocytes [29], the present study implicates relief of constraint from the 14-3-3/p21/p53 pathway in the enhanced proliferative potential of dedifferentiating myocytes.

Recent studies in mice and in zebrafish revealed partial dedifferentiation *in vivo*, evident by disassembly of sarcomeres prior to cell proliferation [7], [8], [17]. We have found that mammalian cardiomyocytes in long-term culture undergo much more extensive dedifferentiation, as evidenced by the loss of typical cardiomyocyte morphology, cardiac filament protein and transcripts, and characteristic electrophysiological properties, with subsequent re-expression of the progenitor cell marker c-kit. The reacquisition of a progenitor/stem cell phenotype in dedifferentiated myocytes was further verified in an *ex vivo* cardiac tissue culture setting by *Cre/LoxP* cell fate mapping of GFP-positive myocytes and their progeny [3], [38], [40], [58], [59]. The orchestrated expressions of cardiac, proliferative, cell cycle and stemness regulatory miRNAs, and related proteins and mRNAs, are consistent with the proposed progenitor/stem cell identity of MDCs. The re-differentiation potential of MDCs further confirms the identity of MDCs as cardiac progenitor/stem cells.

### Limitations

Either of two complementary approaches independently leads to the conclusion that cardiomyocyte dedifferentiation produces CPCs. Both approaches would have to be fundamentally flawed in order to invalidate the concept. Specifically, the starting cardiomyocyte preparation would have to be substantially impure, and the bitransgenic mice would have to be misleading reporters of the cardiomyocyte lineage. We will consider each of these two possibilities in turn.

To purify cardiomyocytes, we adopted multiple sedimentation and Percoll gradient separation steps, and then evaluated purity by various assays. Real-time RT-PCR, capable of detecting 1 contaminating c-kit^+^ cell per 40,000 cardiomyocytes, revealed that nonmyocytes were undetectable in our preparation. An even more sensitive approach, high-density tile scanning confocal microscopy, supported the notion of cardiomyocyte purity, at least within a resolution limit of 1 in 500,000. Given that there is on average one CPC per 10,000 myocytes in the rat heart [2], even a native tissue level of contamination, with no purification, would have fallen well within the detection limit of our methods. Moreover, isolated c-kit^+^ CPCs exhibit very slow proliferative activity *in vitro* [41], making it unlikely that contaminating CPCs in the starting material, even if present, could account for what we have called MDCs.

Genetic cell fate mapping to track cardiomyocytes and their progeny depends on the fidelity of the α-MHC promoter, which has recently been questioned [60]. Here we have shown that the full-length *MYH6* promoter used in the present bitransgenic model drives cardiac-specific gene recombination, resulting in the expression of GFP only in cTnT-positive myocytes as revealed by immunostaining and genotyping, consistent with previous findings [38], [61]–[65]. Richard T. Lee, who created these mice, has stained their hearts for c-kit and found none of the c-kit^+^ cells to be GFP^+^, a result confirmed by flow cytometry (R.T. Lee, personal communication). We find that outgrowth cells from bitransgenic cardiac explants can be c-kit^+^ but GFP^−^ (Fig. 5), further negating an obligatory linkage between c-kit expression and GFP positivity. Thus, the bitransgenic mice appear to label cardiomyocytes (and their progeny) specifically, allowing us to conclude that c-kit^+^/GFP^+^ cells in that model arose from dedifferentiated cardiomyocytes.

The present study shows that cardiomyocyte dedifferentiation and proliferation can occur under highly-artificial conditions *in vitro*, but does not address the crucial follow-up question of how frequently dedifferentiation occurs *in vivo*, and the functional properties of these myocyte-derived cells. Such work, which is necessary to establish the pathophysiological importance of dedifferentiation, would not have been motivated without the demonstration here that spontaneous cardiomyocyte dedifferentiation can occur, and can lead to cells with some key characteristics of resident CPCs.

## Materials and Methods

### Dissociation, Purification, and Primary Culture of Cardiomyocytes

All animal procedures were conducted in accordance with humane animal care standards outlined in the NIH Guide for the Care and Use of Experimental Animals and were approved by the Cedars-Sinai Medical Center Animal Care and Use Committee (IACUC2557, IACUC2424). Cardiomyocytes were isolated from male Wistar-Kyoto rats (5–8 weeks, 70–120 g) by enzymatic dissociation of the whole heart on a Langendorff apparatus as previously described [66]. Heparinized animals were anaesthetized by Ketamine/Xylazine (30 mg/Kg, and 6 mg/Kg, respectively). Hearts were rapidly excised and cleansed to remove blood in ice-cold Tyrode's solution before being mounted to a Langendorff apparatus conjugated to a pressure monitoring device, then perfused retrogradely with the following four oxygenated solutions in sequential order: 1. modified Tyrode's solution containing 1.0 mM Ca^2+^ (2 min), 2. Ca^2+^-free Tyrode's solution (2–3 min), 3. Ca^2+^-free Tyrode's solution containing 0.2 Wünsch unit/mL of collagenase made from Liberase Blendzyme 4 (Roche Molecular Biochemicals, Indianapolis, IN) for 10–15 min depending on digesting conditions, and 4. followed by washing in Kruftbrühe (KB) solution for 5 min. Digested atrium and ventricles were cut off and minced in KB solution, pipetting to dissociate the cells, then filtered through a nylon mesh (200 µm pore size) to remove big pieces of undigested tissues. Isolated cells were rinsed in KB solution and allowed to settle by gravity 3 times to remove debris and non-cardiomyocytes. Resuspended cells in KB solution were loaded above the top layer of a Percoll gradient formed by 20%, 40%, and 70% Percoll and centrifuged at 100x *g* for 20 min to further purify cardiomyocytes. After three washes in KB solution, myocytes were resuspended in KB solution or in culture media for further experiments. Modified Tyrode's solution contained (mmol/L): NaCl 105, KCl 5.4, KH_2_PO_4_ 0.6, NaH_2_PO_4_ 0.6, NaHCO_3_ 6, KHCO3 5, CaCl_2_ 1, MgCl_2_ 1, HEPES 10, glucose 5, taurine 20 (pH 7.35 with NaOH), and KB solution had (mmol/L): KCl 20, KH_2_PO_4_ 10, K^+^-glutamate 70, MgCl_2_ 1, glucose 25, β-hydroxybutyric acid 10, taurine 20, EGTA 0.5, HEPES 10, and 0.1% albumin (pH 7.25 with KOH). The purity of myocyte preparations were evaluated in cells cytospun on to 22 mm cover glasses by fluorescent immunocytochemistry in combination with high density tile confocal scanning and RT-PCR described in later sections. Chemicals were purchased from Sigma except for those specified.

Purified myocytes were resuspended in Medium 199 (Invitrogen, Carlsbad, CA) supplemented with 110 mg/L sodium pyruvate, 0.1 mmol/L β-mercaptoethanol, 100 U/mL penicillin, 100 µg/mL streptomycin, and 5% FBS (Invitrogen) and cultured in laminin-coated 6-well culture plates or 100-mm dishes at a “normal” density of ∼6000 and ∼9000 cells/cm^2^ for ventricular and atrial myocytes respectively, at 37 °C for one hour before washing to remove dead and non-adherent cells, and repeated once. Serum concentration in medium was gradually increased to 10% and 20%. On the second and third day of plating, medium was replaced to remove dead cells, and then maintained for prolonged culture in the presence of bFGF 0.1 ng/ml, TGF-β3 1 ng/ml, and 20% FBS in the basal IMDM medium supplemented with 100 U/mL penicillin, 100 µg/mL streptomycin, and partially changed every 4–5 days. Within the first 2∼3 days, about 20–30% of cells were lost in the normal dense culture and removed by changing the medium; the cell culture usually became stable thereafter. This is similar to previous studies and may reflect the apoptosis of a certain portion of myocytes. Therefore, it is suggested that dedifferentiation functions as a reprogrammed survival process for cardiomyocytes [54]. No AraC or other inhibitors were used in our experiments.

### Cell imaging and Tracking

In order to verify the proliferation of dedifferentiated myocytes, cells were plated at a lower density. Numeric grid-marked coverslips (Bellco Biotechnology, Vineland, NJ) coated with laminin were used to track changes in identified cells during culture, under time-lapse microscopy (Nikon TE-2000E inverted microscope) for continuous analysis, or intermittently in an inverted microscope (Nikon TE-2000 U), with phase contrast objectives. Images were captured with a monochrome CCD camera (Q-Imaging, Surrey, BC, Canada) with program suite Image Pro Plus (Media Cybernetics, Bethesda, MD). A 3CCD Color video camera (Sony) connected to a personal computer was used to capture real-time images and videos of beating cells and spheres.

### Culture of Myocyte-Derived Cells

At 10 days to 2 weeks after the culture, the loosely-adherent myocyte-derived cells (MDCs) were harvested by gentle pipetting 3 times with a disposable transfer pipette. Cells were grown in serum-rich myocyte culture medium for the experiments detecting the markers in fresh isolated cells. Alternatively, MDC culture medium, which was DMEM/F12 supplemented with 0.1 mM β-mercaptoethanol, bFGF 0.1 ng/ml, TGF-β3 1 ng/ml, 100 U/ml penicillin, 100 µg/ml streptomycin, and 10% FBS, was used to maintain the cells in 95% humidity, 5% CO_2_ at 37°C.

### Labeling of Myocytes with BrdU

To study the cell cycle progression, cells were cultured in the presence of 5-bromo-2-deoxyuridine (BrdU; Sigma; 5 µM) for various periods starting after one day of myocyte culture before immunoassays described previously [29]. Briefly, washed cells were fixed with 4% paraformaldehyde and permeabilized with 0.2% Triton-X100, followed by block with 5% normal donkey serum for 1 hour, then incubation with primary and secondary antibodies to detect membrane molecules. Cells were washed in phosphate buffered saline and re-fixed with 4% paraformaldehyde, and incubated in 1N HCl on ice for 10 min, and in 2N HCl at room temperature and 37°C each for 10 min, followed by wash with borate buffer (0.1 M, pH 8.5) at room temperature three times each for 5 min. Cells were then washed in 0.1% Triton-X100 in phosphate buffered saline and re-blocked in 5% normal donkey serum for 30 min at room temperature, then incubated with 10 µg/ml sheep anti-BrdU antibody (Abcam) diluted in blocking buffer for 1.5 hr at room temperature, then washed, and subsequently incubated with Alexa Fluor 647-conjugated donkey anti-sheep secondary antibody to detect cells with BrdU incorporation.

### Fluorescent Immunocytochemistry

Cellular phenotypes were analyzed as previously described using fluorescent immunocytochemical assays [4], [66]. To test the expression of stem cell markers, rabbit polyclonal antibody (pAb) against c-kit (CD117) (Santa Cruz Biotechnology, Santa Cruz, CA) or CD34 (Abcam, Cambridge, MA), mouse monoclonal antibody (mAb) against Sca-1 (Invitrogen), and goat pAb against Thy-1 (CD90) were used as primary antibodies. Expression of cardiac markers was assessed using antibodies including mouse mAb for α-MHC from Abcam or Sigma, mouse mAb cTnT, and rabbit pAb Cx43 and CD31 from Invitrogen. Primary antibodies against cell cycle-specific molecules: Ki67, Histone H3 (phosphor-S10) (H3P) and anti-BrdU were from Abcam. Goat polyclonal antibody against 14-3-3η was from Santa Cruz Biotechnology, and rabbit polyclonal p21 antibody and mouse monoclonal p53 antibody were from Abcam. Chicken anti-GFP polyclonal antibody was from Abcam. The specificity of antibodies was confirmed by blocking peptides or control cells. Donkey anti-mouse, anti-rabbit, or anti-goat, or goat-anti chicken antibodies with Alexa Fluor conjugates were used as secondary antibodies.

Direct immunostaining was also performed to test the expression of stem cell markers in freshly harvested MDCs using PE-conjugated mouse mAbs against c-kit (BD Biosciences), or FITC-conjugated CD90 (Abcam).

In MDC-formed spheres, stem cell and cardiac markers were detected using whole-mount immunofluorescent techniques and examined with standard and Z-stack confocal laser scan microscopes (from Zeiss or Leica) [4]. Signals of samples from fixation by either paraformaldehyde or cold acetone/methanol were verified and auto-fluorescence was excluded. The acquisition settings were optimized to avoid false positive or false negative signal. To evaluate the purity of myocyte preparation, we used Tile Scanning Function (Leica Confocal) to examine the whole cover glass (22 mm diameter) after cells were subjected to immunostaining. Images were processed by Zeiss LSM 510 or Leica LAS software suites, and molecule expression levels were semi-quantified by their fluorescence as described before [66]–[68].

### Patch-Clamp Recordings

Electrophysiological properties characteristic to cardiomyocytes in normal or dedifferentiated myocytes at 4 and 7 days and in semi-adherent myocyte-derived cells (MDCs) were evaluated by whole-cell patch techniques as described previously [66], [69]. Inward rectifier potassium current (I_K1_) was recorded in voltage-clamp mode. Resting membrane potential and cell capacitance were also recorded as described [66], [69].

### RT-PCR

Reverse-transcription Polymerase Chain Reaction (RT-PCR) was performed to test the mRNA expression of both stem cell and cardiac markers. Extraction of total RNA from rat heart tissue, bone marrow cells flushed from femurs, purified myocytes, MDC, and MDC spheres, and one-step RT-PCR were carried out with commercially available kits (Qiagen, Valencia, CA). Primer pairs for *c-kit*, *sca-1*, *CD90*, *CD31*, *CD34*, *α-MHC*, *GATA4*, *Nkx2.5*, and *β-actin* are listed in Supplemental Table S1. Additionally, one-step real-time RT-PCR was performed to estimate the detection limit on c-kit^+^ cells sorted from bone marrow cells and serial diluted and mixed with c-kit^-^ bone marrow cells or with purified myocyte preparation.

### MicroRNA Assays in Cardiomyocytes and Myocyte-Derived Cells (MDCs)

Total RNA including MicroRNA (miRNA) was isolated from purified cardiomyocytes or MDCs using a mirVana Paris kit (Ambion). TaqMan Rodent MicroRNA Set A Array v2.0 which contains 384 TaqMan MicroRNA Assays was used to detect the expression levels of miRNAs. miRNA in 300 ng total RNA was first reverse-transcribed into cDNA using MegaPlex RT primer pool set A and miRNA RT kit, followed by pre-amplification of cDNA with MegaPlex PreAmp primers pool set A and TaqMan PreAmp master mix. cDNA was diluted and mixed with TaqMan universal master mix before loaded into the pre-configured micro fluidic card of miRNA. Real-time reaction was run on a 7900HT Fast Real-Time PCR System (Applied Biosystems) and data collected and analyzed using the accompanied SDS 2.3 software. Two biological repeats and two technical repeats were performed. Ct values were normalized to endogenous miRNA control Mamm U6, and comparative 2^−ΔΔCt^ method was used to evaluate the fold change of miRNAs in MDCs *vs.* cardiomyocytes [70], [71].

### Lentiviral Transduction of Cardiac Reporter

MDC-formed spheres were transduced with the third generation lentivirus expressing eGFP under the control of cardiac *α-MHC* promoter as described previously [20].

### Genetic Cell Fate Mapping with Bitransgenic MerCreMer/ZEG Mouse Cardiomyocytes

Bi-transgenic MerCreMer/ZEG mice were produced by crossbreeding cardiomyocyte-specific MerCreMer mice [39] and ZEG mice [40] (Jackson Laboratory) as described previously [38]. The ZEG reporter mouse carries cytomegalovirus (CMV) enhancer/chicken *β-actin* promoter driving floxed β-galactosidase and multiple stop codons, followed by eGFP. Animal genotype was verified by standard PCR on tail genomic DNA using the following primers: MerCreMer forward: 5′- ATACCGGAGATCATGCAAGC-3′; MerCreMer backward: 5′- AGGTGGACCTGATCATGGAG-3′; and ZEG forward: 5′- ACGGCAAGCTGACCCTGAAG-3′; ZEG backward: 5′- AAGATGGTGCGCTCCTGGAC-3′; internal control (IL2) forward: 5′- CTAGGCCACAGAATTGAAAGATCT-3′; and internal control backward: 5′- GGATGATGCTAGAATTTCCACCTAC-3′. Double heterozygous bitransgenic MerCreMer-Z/EG mice were used for the cell fate tracing experiments after induction of Cre recombination for GFP labeling in cardiomyocytes by 4-OH-Tamoxifen treatment. 4-OH-tamoxifen (Sigma), dissolved in peanut oil (Sigma) at a concentration of 5 mg/ml, was intraperitoneally injected into 4–6 week-old MerCreMer-Z/EG mice daily at a dosage of 0.5 mg/d. Fourteen consecutive injections of 4-OH-tamoxifen showed significant GFP labeling of overall cardiomyocytes, as also reported previously [38]. Cardiac-specific gene recombination (labeling of GFP to cardiomyocytes) was verified by both genotyping in purified GFP-positive myocytes (isolated similarly as for rat myocytes) and GFP-positive or negative MDCs from tissue culture [20], and by immunostaining of cardiac marker cTnT in isolated myocytes and heart tissues. Tamoxifen-treated bitransgenic mice were used at the age of 6–10 week and we did not see significant difference in their capability of dedifferentiation and proliferation, or regaining of cardiac stem cell phenotypes.

Dissociated transgenic mouse myocytes are difficult to maintain in long-term culture. We employed established explant culture techniques [4], [72] with modifications to attain better viability of cardiomyocytes in culture. Briefly, mouse hearts were perfused similarly as for the rat myocyte protocol, with Ca^2+^-free Tyrode's solution for 2 min, followed by digestion for 6–8 min in Ca^2+^-free Tyrode's solution containing 0.15 Wünsch unit/mL of collagenase made from Liberase Blendzyme 4, followed by washing in KB solution for 5 min. The hearts were cut into small pieces in ∼1 mm^3^ and rinsed in KB solution for 3 times. Tissues were transferred to laminin-coated 10 mm tissue culture dishes or 2-well chamber CultureSlides (BD Biosciences), with M199 medium containing 100 U/mL penicillin, 100 µg/mL streptomycin, and 5% FBS (Invitrogen) and supplements of 25 µM Blebbistatin, ITS (5 µg/ml insulin and transferrin, 5 ng/ml selenium), and 10 mM β-hydroxybutyric acid for the first two days of culture. Blebbistatin, ITS, and β-hydroxybutyric acid were replaced with bFGF 0.1 ng/ml and TGF-β3 1 ng/ml, and FBS increased to 20% in subsequent cultures. Medium was partially replaced every 4–5 days.

### Statistics

Data were expressed as mean ± SEM, and un-paired Student t-test were used to evaluate the significance of differences between groups, with a *p*<0.05 considered as significantly different.

## Supporting Information

Table S1 Primers used for RT-PCR detection.
(0.08 MB PDF)

Figure S1 Purity of cardiomyocyte preparation. Myocyte preparation was cyto-spun onto laminin-coated 22 mm cover-glass and subjected to fluorescent immunostaining. Shown are example composite images of high density tile scanning confocal images. Scanning of the full preparation reveals the absence of any non-myocyte marker. Cardiomyocyte preparations are positive to cTnT (green, A) or α-MHC (green, B–D), but not to stem cell marker c-kit (red, A), or endothelial cell marker CD31 (red, B), or endothelial progenitor marker CD34 (red, C), or fibroblast marker CD90 (red, D). Top panels are magnified views of the green squared scanning region.
(8.20 MB PDF)

Figure S2 Evaluation of the detection limit of RT-PCR. A, Standard curve constructed by plotting the Cts with the numbers of serially-diluted c-kit^+^ cells bone marrow cells. B, Amplification plots of c-kit in cell mixtures (indicated number of c-kit^+^ cells mixed with 40,000 cardiomyocytes) with 1, 2, 5, or 10 c-kit^+^ bone marrow cells.
(0.79 MB JPG)

Figure S3 Mitosis and Cytokinesis of Tracked Cardiomyocytes. Example confocal images for the expressions of Ki67 (green), Histone 3 phospho-S10 (H3P; red) in dedifferentiated myocytes counterstained with DAPI for nuclei (blue). Panel a shows one myocyte (on right) at G0 phase, without expression of Ki67 or H3P, and the other myocyte (on left) at interphase with both molecules expressed. *b*, *c*, and *d*: cell at prophase, anaphase, and telophase, respectively. *b* and *d* also show cells at resting state without Ki67 or H3P expression.
(1.08 MB JPG)

Figure S4 Time for the first confluence of myocyte culture (A), MDC diameter (B), and time required for sphere (Sp) to beat (C).
(0.21 MB JPG)

Figure S5 Green fluorescence in a beating MDC sphere (please see Online Movie S5) transduced with replication-defective lentivirus encoding eGFP driven by cardiac α-MHC (*MHY6*) promoter at 3d. Scale bar, 100 µm.
(0.51 MB JPG)

Movie S1 MDC-formed sphere loosely adherent to the culture layer beats spontaneously.
(0.77 MB WMV)

Movie S2 MDC-formed sphere loosely adherent to the culture layer beats; shown is in higher magnification to reveal the spontaneous activity.
(1.02 MB WMV)

Movie S3 MDC-formed sphere beats in suspension.
(2.97 MB WMV)

Movie S4 MDC-formed sphere cultured on slide continues to exhibit spontaneous activity.
(1.07 MB WMV)

Movie S5 Spontaneous contraction of a MDC-formed sphere transduced with α-MHC-eGFP lentivirus (see Figure S5).
(3.25 MB WMV)
