# Supplementary material for: Dedifferentiation and Proliferation of Mammalian Cardiomyocytes
Source: PLoS One. 2010 Sep 3;5(9):e12559. doi: 10.1371/journal.pone.0012559 (PMC2933247; doi:10.1371/journal.pone.0012559)
Supplement: Table S1 — Primers used for RT-PCR detection. (0.08 MB PDF) [file pone.0012559.s001.pdf]

Table S1. Primers used for RT-PCR detection.

Molecule	Access #	Primer		Product		
		sense oligo 5' ….3'	antisense oligo 5'…..3'	start	stop	length
c-Kit	NM_022264	AGCCGTCTCCACCATCCATCCAG	GCGGACCAGTGCGTCGTTGTCTT	142	449	308
Sca-1	XM_343263	CATCTTTCTCCTGGCCCTACT	GAGGACTGAGCCCAGGATGAA	46	390	345
CD90/Thy1	NM_012673	CCTGCCTGGTGAACCAGAACCTT	GCAGGCTTATGCCACCACACTTG	125	451	327
CD31	NM_031591	AGAAGGAAGAGACGGTGTTG	TTAGGAGGCGGTAAGTGATG	1241	1498	258
CD34	XM_001070343	TCAGAGACCACGGTCAACTT	ACTCCTCGGATTCCTGAACA	417	721	305
GATA4	NM_144730	TCTAAGACACCAGCAGGTCCTC	TTGGAGCTGGCCTGTGAT	1540	1823	284
Nkx2.5	NM_053651	TTATCCGCGAGCCTACGGTGA	CTGCCGCTGTCGCTTACACTT	366	684	319
α-MHC	NM_017239	AGTCAGAGAAGGAGCGCCTA	TAGATCATCCAGGCCGCATA	87	378	292
β-actin	NM_031144.2	ATATCGCTGCGCTCGTCGTC	CGTCCCAGTTGGTGACAATG	92	322	231
