# Supplementary figures and images for: Dedifferentiation and Proliferation of Mammalian Cardiomyocytes
Source: PLoS One. 2010 Sep 3;5(9):e12559. doi: 10.1371/journal.pone.0012559 (PMC2933247; doi:10.1371/journal.pone.0012559)

**A**

**cTnT**

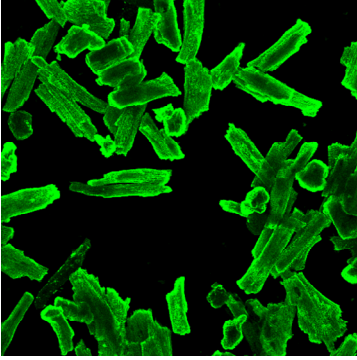

**c-kit**

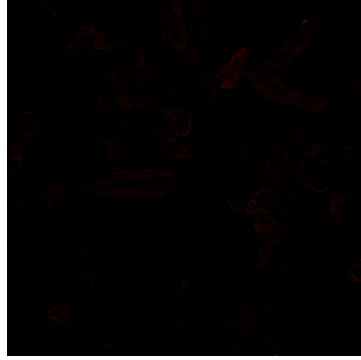

**phase**

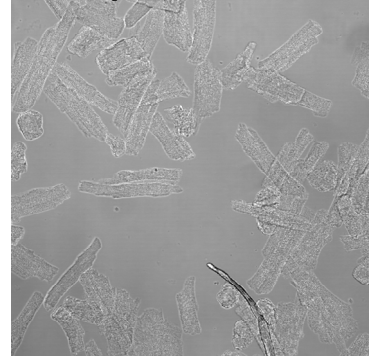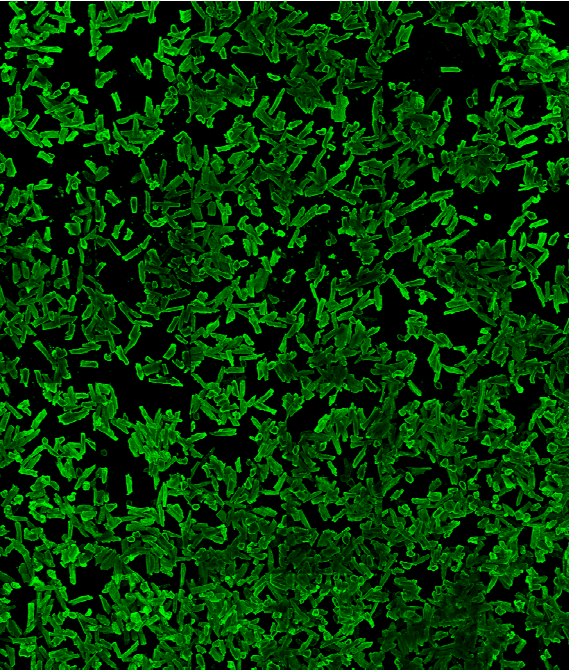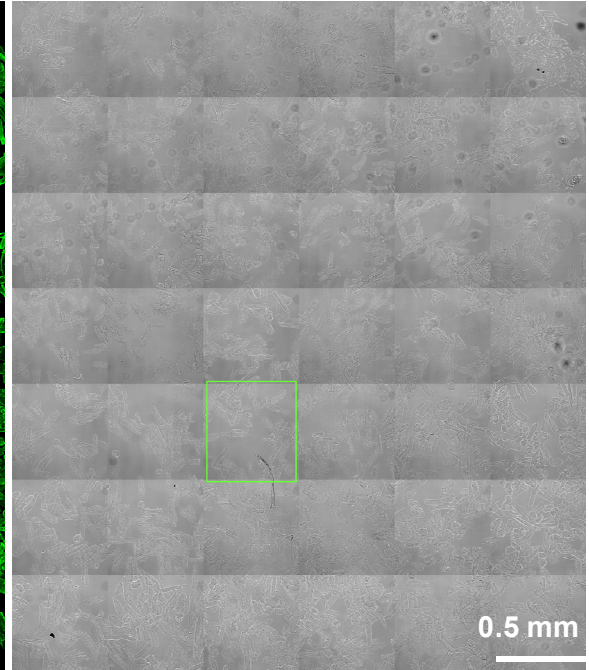

**B**

$\alpha$ -MHC

CD31

phase

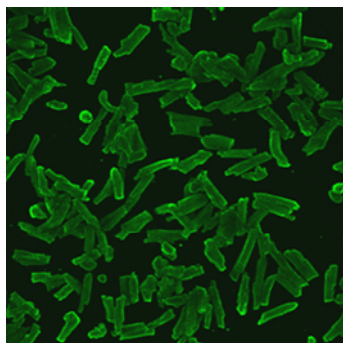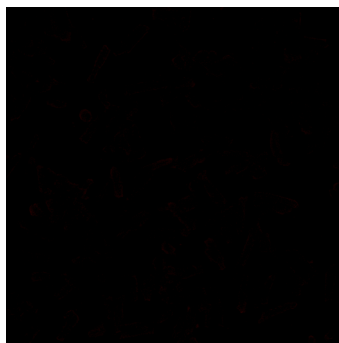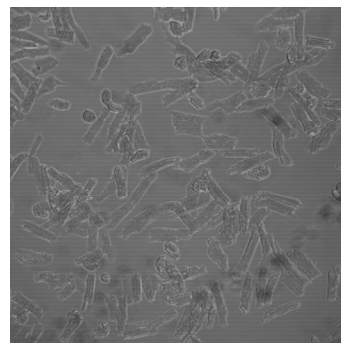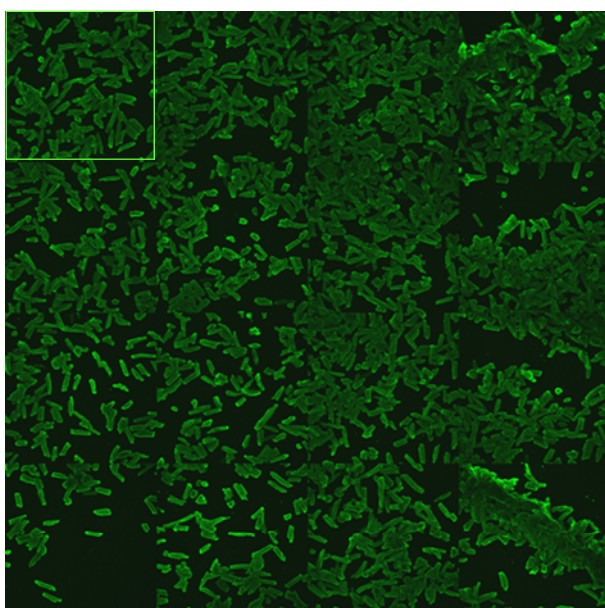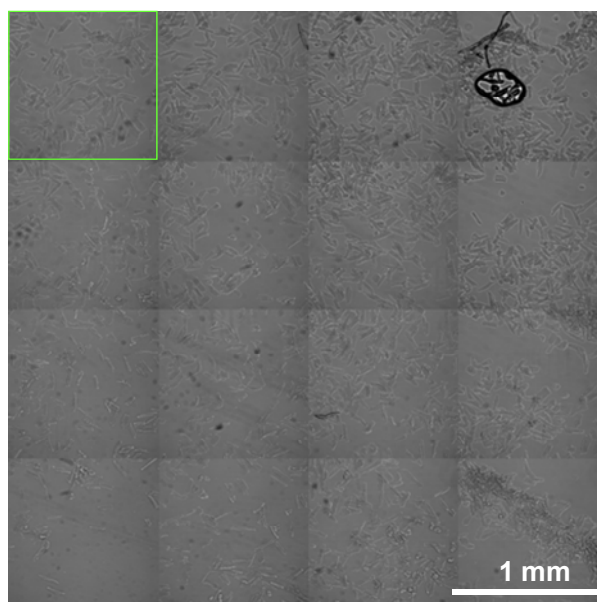

C

$\alpha$ -MHC

CD34

phase

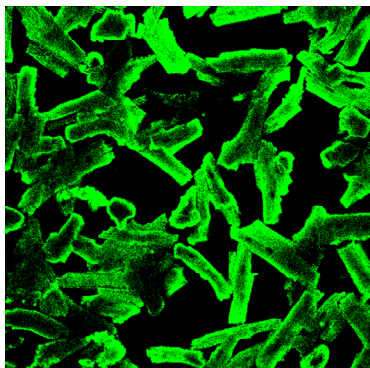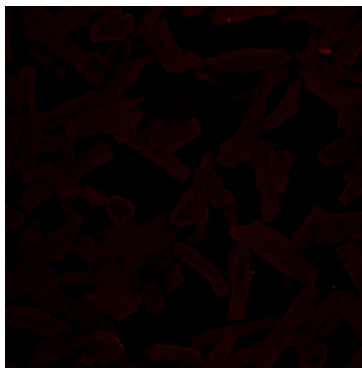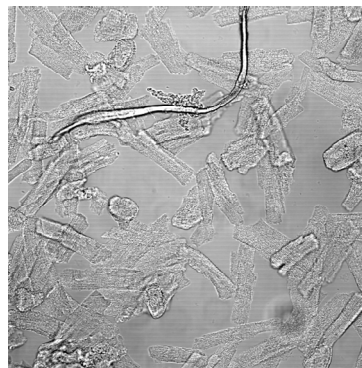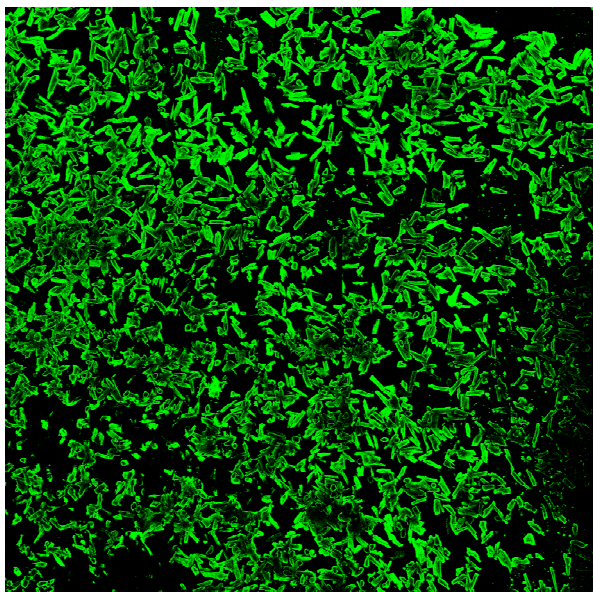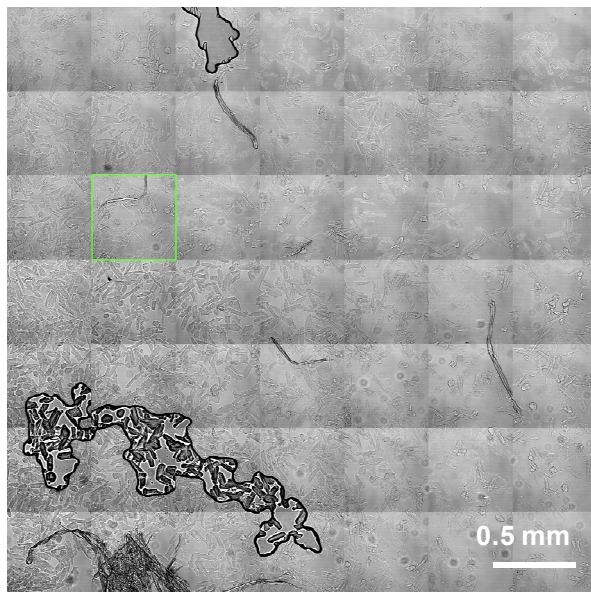

D

$\alpha$ -MHC

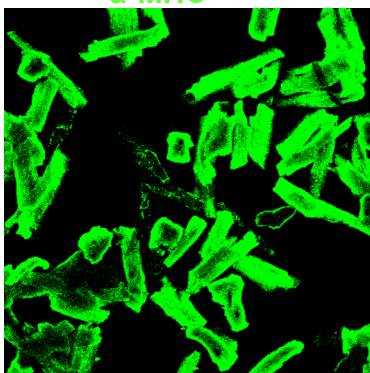

CD90

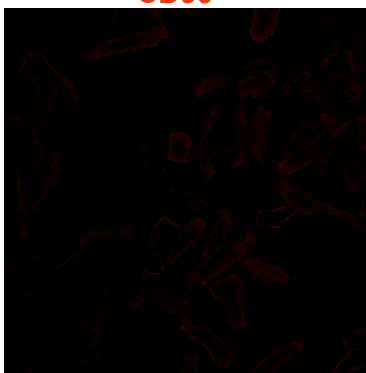

phase

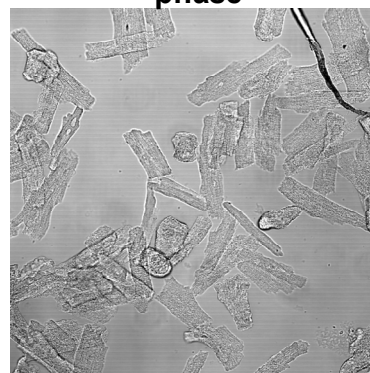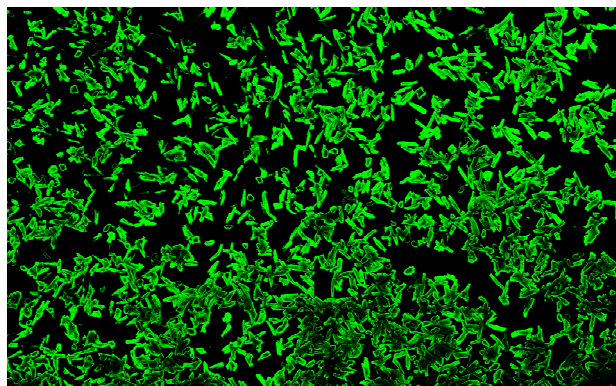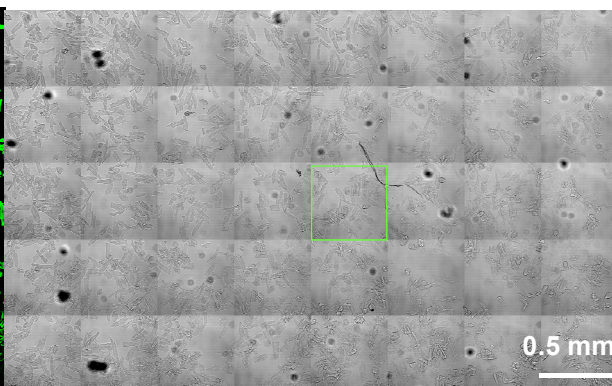

Supplement: Figure S1 — Purity of cardiomyocyte preparation. Myocyte preparation was cyto-spun onto laminin-coated 22 mm cover-glass and subjected to fluorescent immunostaining. Shown are example composite images of high density tile scanning confocal images. Scanning of the full preparation reveals the absence of any non-myocyte marker. Cardiomyocyte preparations are positive to cTnT (green, A) or α-MHC (green, B–D), but not to stem cell marker c-kit (red, A), or endothelial cell marker CD31 (red, B), or endothelial progenitor marker CD34 (red, C), or fibroblast marker CD90 (red, D). Top panels are magnified views of the green squared scanning region. (8.20 MB PDF) [file pone.0012559.s002.pdf]

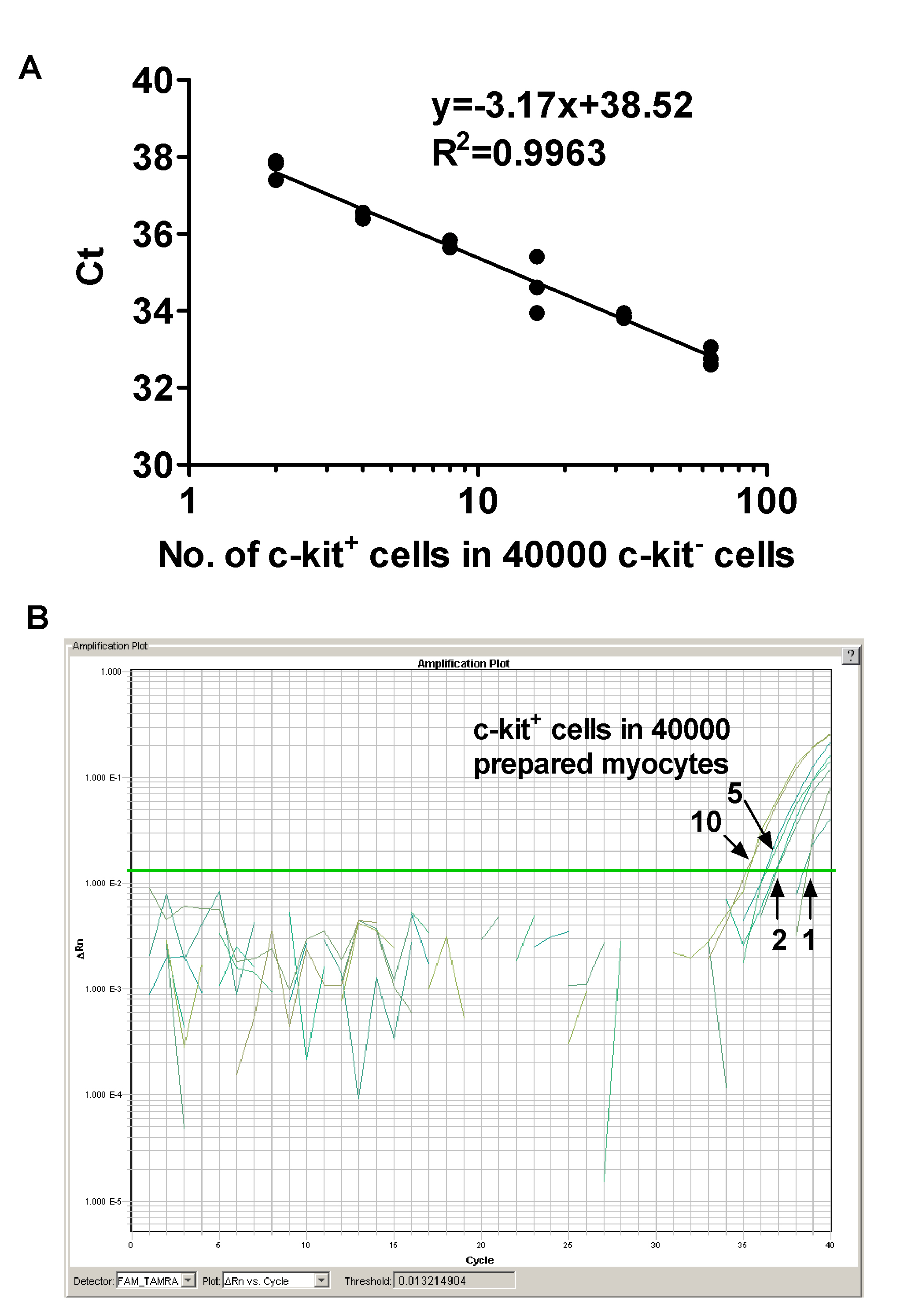

Supplement: Figure S2 — Evaluation of the detection limit of RT-PCR. A, Standard curve constructed by plotting the Cts with the numbers of serially-diluted c-kit+ cells bone marrow cells. B, Amplification plots of c-kit in cell mixtures (indicated number of c-kit+ cells mixed with 40,000 cardiomyocytes) with 1, 2, 5, or 10 c-kit+ bone marrow cells. (0.79 MB JPG) [file pone.0012559.s003.jpg]

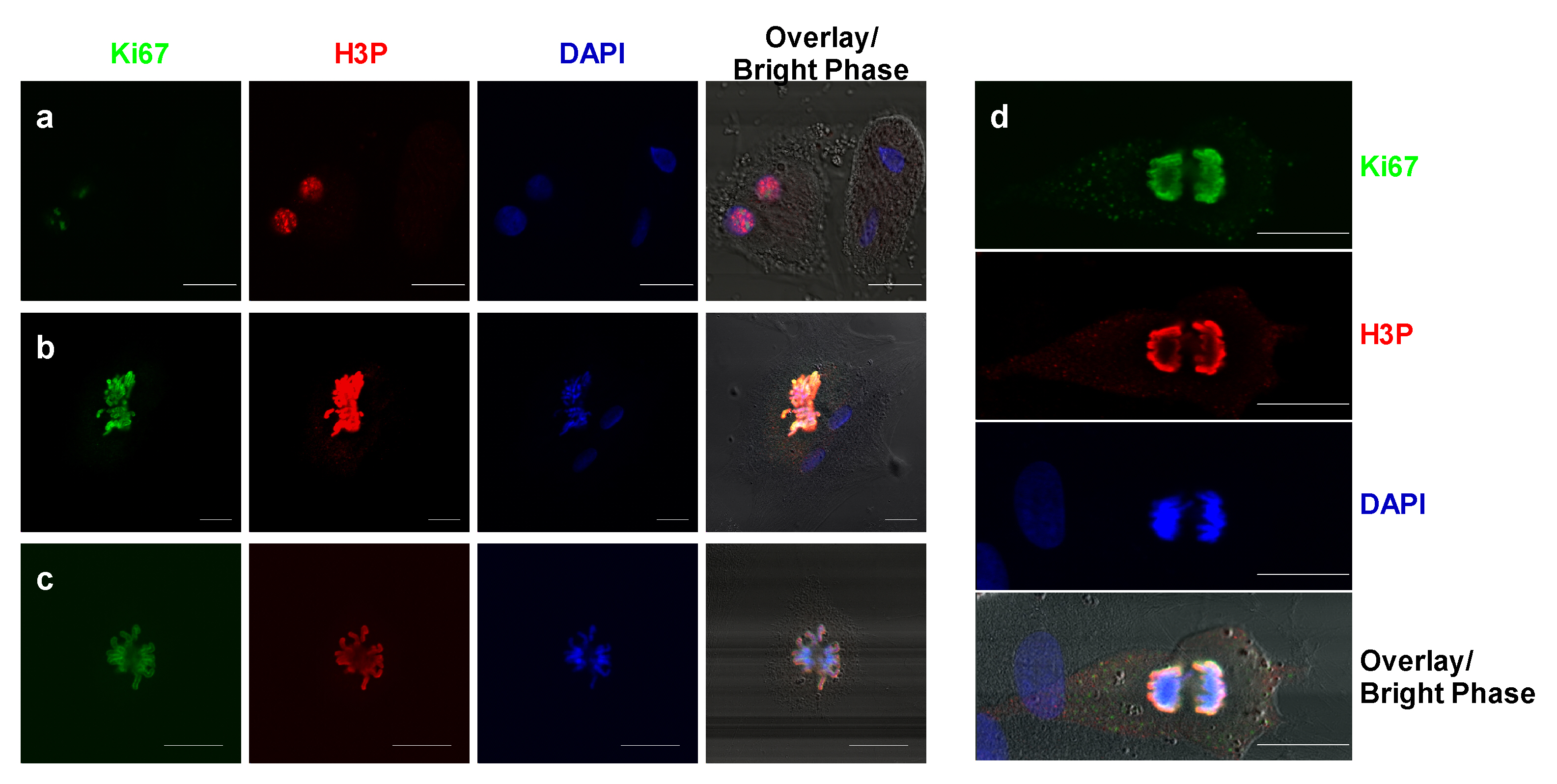

Supplement: Figure S3 — Mitosis and Cytokinesis of Tracked Cardiomyocytes. Example confocal images for the expressions of Ki67 (green), Histone 3 phospho-S10 (H3P; red) in dedifferentiated myocytes counterstained with DAPI for nuclei (blue). Panel a shows one myocyte (on right) at G0 phase, without expression of Ki67 or H3P, and the other myocyte (on left) at interphase with both molecules expressed. b, c, and d: cell at prophase, anaphase, and telophase, respectively. b and d also show cells at resting state without Ki67 or H3P expression. (1.08 MB JPG) [file pone.0012559.s004.jpg]

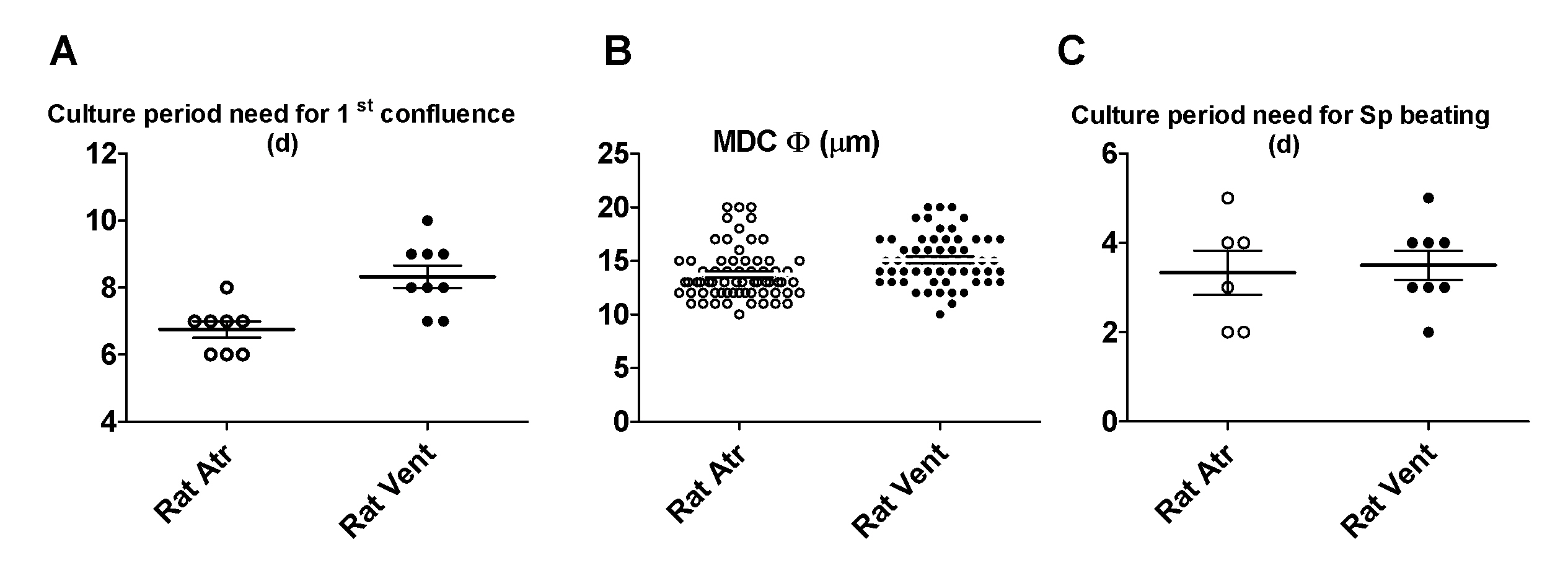

Supplement: Figure S4 — Time for the first confluence of myocyte culture (A), MDC diameter (B), and time required for sphere (Sp) to beat (C). (0.21 MB JPG) [file pone.0012559.s005.jpg]

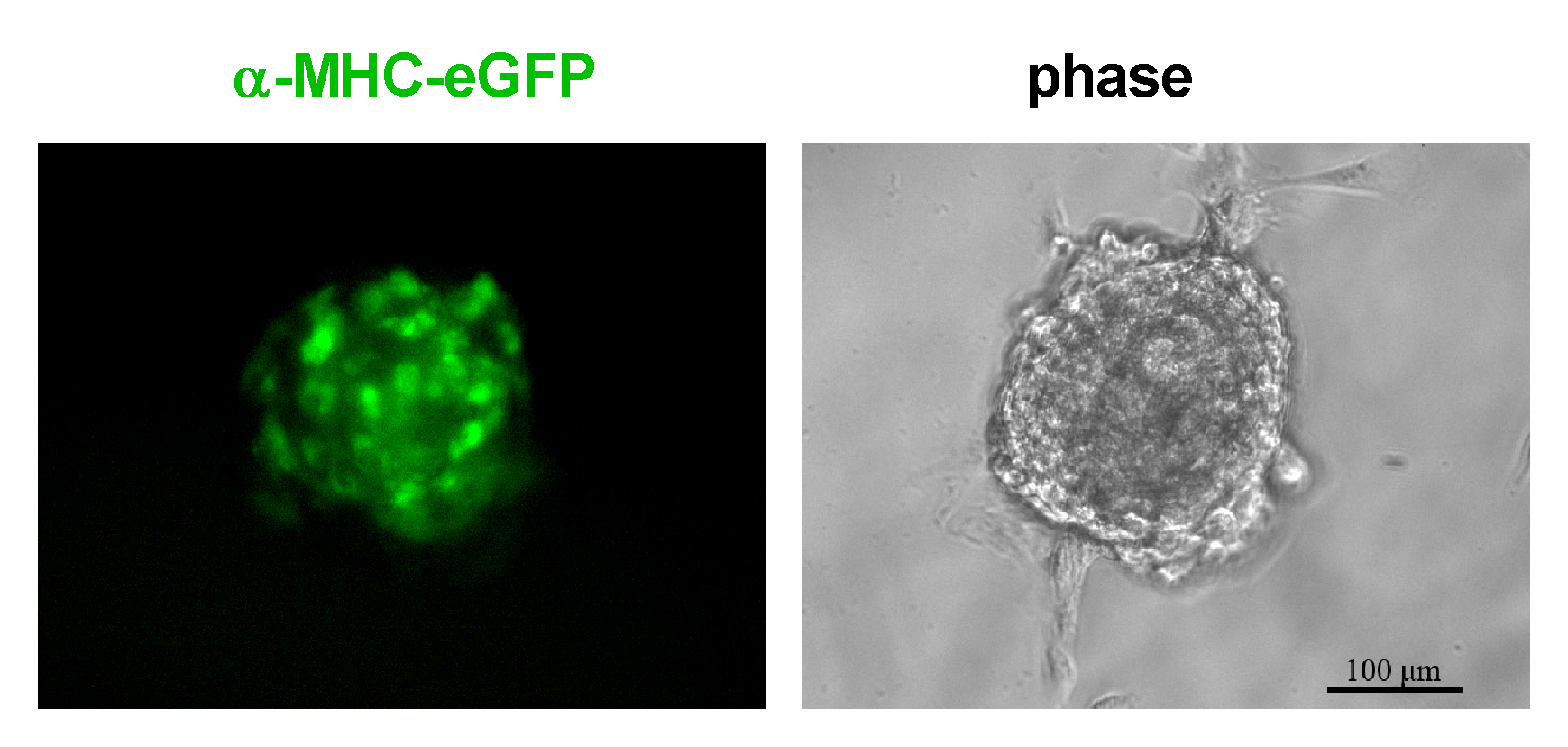

Supplement: Figure S5 — Green fluorescence in a beating MDC sphere (please see Online Movie S5) transduced with replication-defective lentivirus encoding eGFP driven by cardiac α-MHC (MHY6) promoter at 3d. Scale bar, 100 µm. (0.51 MB JPG) [file pone.0012559.s006.jpg]
